# Combinations of Antibiotics Effective against Extensively- and Pandrug-Resistant *Acinetobacter baumannii* Patient Isolates

**DOI:** 10.3390/microorganisms12071353

**Published:** 2024-07-02

**Authors:** Justin Halim, Rachel A. Carr, Rebecca Fliorent, Keertana Jonnalagadda, Maftuna Kurbonnazarova, Muskanjot Kaur, Ian Millstein, Valerie J. Carabetta

**Affiliations:** 1 Department of Biomedical Sciences, Cooper Medical School of Rowan University, Camden, NJ 08103, USA; halimj77@rowan.edu (J.H.); carrr@rowan.edu (R.A.C.); millst89@rowan.edu (I.M.); 2 Rowan-Virtua School of Osteopathic Medicine, Stratford, NJ 08084, USA; fliore92@rowan.edu (R.F.); jonnal56@rowan.edu (K.J.); kurbon64@rowan.edu (M.K.); kaurmu89@rowan.edu (M.K.)

**Keywords:** multidrug-resistant, omadacycline, eravacycline, rifampin, minocycline, MDR, PDR, XDR, bacteria, antibiotic resistance

## Abstract

Infections due to drug-resistant *Acinetobacter baumannii* strains are increasing and cause significant morbidity and mortality, especially in hospitalized and critically ill patients. *A. baumannii* rapidly develops resistance to numerous antibiotics, and antibiotics traditionally used against this deadly pathogen have been failing in recent years, highlighting the need to identify new treatment strategies. Treatment options that have shown promise include revisiting common antibiotics not typically used against *A. baumannii*, evaluating new antibiotics recently introduced to market, and identifying combinations of antibiotics that display synergistic interactions. In this study, we characterized the antibiotic susceptibility profiles of extensively (XDR) and pandrug-resistant (PDR) *A. baumannii* patient isolates. We examined the potency of 22 standard-of-care antibiotics and the newer antibiotics eravacycline, omadacycline, and plazomicin against these strains. Furthermore, we examined combinations of these antibiotics against our collection to identify synergistic effects. We found that this collection is highly resistant to most or all standard-of-care antibiotics, except for minocycline and rifampin. We show that eravacycline and omadacycline are effective against these strains based on minimum inhibitory concentrations. We also identified two highly effective combinations, cefepime and amikacin and cefepime and ampicillin–sulbactam, which exhibited high rates of synergy against this collection. This information is valuable in our battle against highly drug resistant and virtually untreatable *A. baumannii* infections.

## 1. Introduction

*Acinetobacter baumannii* is an aerobic, Gram-negative coccobacillary bacteria that is an opportunistic pathogen in humans [1,2]. They frequently are hospital-associated infections, particularly those involving mechanical ventilation, central venous catheterization, urinary catheterization, and intensive care unit (ICU) admission or prolonged stays [2,3]. The United States National Healthcare Safety Network (NHSN) reported that *A. baumannii* accounted for 0.4% of all reported hospital-acquired infections in the U.S. from 2018 to 2021 [4]. Furthermore, *A. baumannii* accounted for 19% of ICU infections in Asia, and 19–50% of nosocomial pneumonia cases in Asian, Latin American, and Middle Eastern countries [5,6,7]. Indeed, nosocomial *A. baumannii* infections exhibit high mortality rates, with previous studies showing crude mortality rates ranging from 40% to 80% [8,9].

*A. baumannii* poses a significant threat due to its high prevalence of antibiotic resistance [10]. They can become drug-resistant via various mechanisms, including increasing expression of chromosomal efflux systems, expressing aminoglycoside modifying enzymes (AMEs) and beta-lactamases, and altering the level or structure of penicillin-binding proteins [2,10]. Antimicrobial resistance has dramatically risen within the last few decades, with the overall percentage of multidrug-resistant (MDR) isolates making up 40% to 80% of total clinical isolates, as estimated by the Center for Disease Control and Prevention (CDC) from 2011–2014 [11]. MDR is defined as non-susceptibility to at least one agent in three or more antimicrobial classes [12]. While MDR *A. baumannii* strains have long been documented, in recent years, there has also been increasing emergence of extensively drug-resistant (XDR) strains, which are non-susceptible to at least one antibiotic agent in all but two or fewer antimicrobial classes, and pandrug-resistant (PDR) strains, which are non-susceptible to all agents in all standard-of-care antimicrobial categories [11,13]. Currently, the carbapenem drugs imipenem and meropenem are effective first-line treatment options for MDR *A. baumannii* infections. Additionally, polymyxins, a class of cell membrane-disrupting antimicrobials, and tigecycline, a third-generation tetracycline, are used as last-line agents for severe MDR infections. However, there has been an increased prevalence of carbapenem-resistant *A. baumannii* (CRAB) isolates, defined by the Clinical and Laboratory Standards Institute (CLSI) as resistant to both imipenem and meropenem (minimum inhibitory concentration (MIC) ≥ 8 μg/mL) and with increased resistance to both polymyxins and tigecycline [14,15,16,17,18]. One surveillance study found that resistance rates to carbapenems among *A. baumannii* increased from 21.0% in 2003 to 47.9% in 2012, while resistance rates to colistin increased from 2.8% in 2006 to 6.9% in 2012 [15]. Furthermore, polymyxin drugs, particularly colistin, have fallen out of favor due to their considerable risk for adverse effects such as nephrotoxicity and neurotoxicity [19]. Since 2022, the CLSI has stopped reporting a susceptible cutoff value for *A. baumannii* for colistin and polymyxin B due to limited clinical efficacy [18]. Given the increasing antimicrobial resistance to these established drugs, it is crucial that new therapeutic strategies be identified to confront the rising rates of XDR and PDR *A. baumannii* infections.

One promising avenue for identifying new treatment options for highly drug-resistant *A. baumannii* infections has been the introduction of new antibiotics to the market. Eravacycline is a fluorocycline drug in the tetracycline class that was approved for the treatment of complicated intra-abdominal infections caused by various Gram-negative organisms, such as *Escherichia coli*, *Klebsiella pneumoniae*, *Citrobacter freundii*, *Enterobacter cloacae*, and *Klebsiella oxytoca*, Gram-positive organisms, including methicillin-resistant *Staphylococcus aureus* (MRSA)*, Streptococcus anginosus* group, *Enterococcus faecalis*, and *E. faecium*, and anaerobic species, like *Clostridioides perfringens* and *Bacteroides* species [20]. Although not formally approved for *Acinetobacter* spp., prior research has demonstrated that eravacycline exhibits promising in vitro activity against *A. baumannii* isolates [21,22]. Omadacycline is an aminomethylcycline antibiotic that was approved for the treatment of community-acquired bacterial pneumonia and acute bacterial skin and skin structure infections, and has been shown to be effective against *A. baumannii* both in vitro and clinically [23,24]. Plazomicin is a new aminoglycoside approved for the treatment of complicated urinary tract infections, and it also possesses in vitro antimicrobial activity against *A. baumannii* [25,26]. Plazomicin has a similar structure to the aminoglycoside gentamicin but contains synthetic modifications that allow evasion from AMEs [27]. However, available data regarding the efficacy and clinical utility of these new drugs against highly drug resistant *A. baumannii* strains is lacking.

With increasing rates of resistance to all drug classes, identifying combinations of existing, Food and Drug Administration (FDA)-approved drugs that exhibit combinatorial effects is another promising strategy to treat *A. baumannii* infections. Antimicrobial synergism is defined as a combinatorial effect in which two or more antimicrobials produce an effect that is more potent than if each antimicrobial was evaluated individually [28]. Previous studies have identified effective antibiotic combinations that display in vitro synergistic activity against MDR *A. baumannii*, which primarily focused on standard-of-care drugs, particularly colistin. Numerous studies have demonstrated significant in vitro synergistic effects between colistin and carbapenems, with a particular emphasis on meropenem [29,30,31,32]. Despite this, one clinical trial found that colistin–carbapenem combination therapies were not superior to colistin monotherapy in treating *A. baumannii* bacteremia, ventilator-associated pneumonia, hospital-acquired pneumonia, or urosepsis [33]. With regard to the carbapenems, previous studies have demonstrated in vitro synergistic effects of meropenem when combined with tigecycline [34,35], ceftazidime [36], rifampin [34], and sulbactam [34,37]. Additionally, ampicillin–sulbactam exhibits synergistic effects when combined with amikacin [38] and levofloxacin [39]. In general, there are limited studies regarding synergistic effects of newer antibiotics in combination with standard-of-care drugs. One study reported synergistic effects of eravacycline combined with either ceftazidime or imipenem against CRAB isolates [40]. Previously, our lab demonstrated synergism with eravacycline when combined with amikacin against XDR isolates [41]. Clearly, further research exploring other antimicrobial combinations and their clinical efficacy is needed. Here, we characterized the antibiotic susceptibility profiles of 21 highly drug-resistant *A. baumannii* patient isolates against 22 standard-of-care drugs and the newer drugs eravacycline, omadacycline, and plazomicin. In addition, we explored different combinations of old and new drugs, with the goal of identifying novel synergistic or additive combinations. All this information will be valuable in our battle against highly drug resistant and virtually untreatable *A. baumannii* infections.

## 2. Materials and Methods

### 2.1. Bacterial Strains, Media, and Growth Conditions

The 21 *A. baumannii* isolates were collected during diagnostic workup at Cooper University Hospital (CUH) in Camden, NJ, during a time of increased drug-resistant infections, which occurred from the end of 2018 through 2019, with strains collected from wounds, blood, sputum, tracheal aspirates, or urine. The isolates were deidentified of all patient information. As the project did not involve living individuals or their private identifiable data, the Cooper Human Research Protection Program determined that this project did not require IRB review. Note that strain M15, which was part of our original collection and analysis, was not *A. baumannii,* so it was excluded from the study. Liquid Mueller-Hinton broth (MHB) and Mueller-Hinton agar (MHA) were prepared in-house following standard protocols. Strains were inoculated into MHB and grown overnight in a 37 °C, with shaking. Bacterial growth was monitored by measuring the optical density at 600 nm (OD_600_). Plazomicin was purchased from TOKU-E (Bellingham, WA, USA). Omadacycline was provided by Paratek Pharmaceuticals (Boston, MA, USA). All other antibiotics were purchased as described previously [41].

### 2.2. Determination of the MIC

The MICs of the 22 standard-of-care antibiotics were determined using broth microdilution according to standard protocols freely available from the American Society for Microbiology (ASM) and CLSI. Following overnight growth, cells were diluted into fresh MHB at a starting OD_600_ of 0.05. Each antibiotic to be tested was placed in the first well of a row in a flat-bottom, 96-well plate at a 2X concentration, with the “X” starting values used as previously described [41]. The X-values for the β-lactamase and β-lactam combinations were as follows: ampicillin–sulbactam-128/64, piperacillin–tazobactam 512/16, and ticarcillin–clavulanate 512/34. The X-value for plazomicin was 32. Two-fold serial dilutions were then performed along each row of the plate, and an equal volume of diluted cells was subsequently added into each well. The plates were then incubated overnight in a 37 °C incubator without shaking, and OD_600_ values were read using a Synergy H1 Microplate reader (Biotek) the following day. MIC determinations were made for at least two independent, biological replicates, and values reported represent averages.

### 2.3. Screen for Combinatorial Effects by Disc Diffusion

Disc diffusion susceptibility testing was then performed in accordance with the ASM’s disc diffusion protocol, which provides a detailed version of the standard CLSI protocol [42]. Overnight cultures were diluted into 2 mL of fresh MH broth and allowed to grow at 37 °C for 2–3 h. Following the pre-growth, 300 μL of culture was spread onto a 150 mm × 15 mm MHA plate. Commercially available discs containing tetracycline, trimethoprim–sulfamethoxazole, levofloxacin, ceftriaxone, and piperacillin–tazobactam were used. For all other antibiotics tested—meropenem, ampicillin–sulbactam, amikacin, minocycline, tigecycline, omadacycline, eravacycline, and rifampin—filter paper discs 6 mm in diameter were prepared by adding a specific amount of drug according to CLSI recommendations as previously described [41] and allowing them to dry for at least one hour at 30 °C. The disc diffusion assays were used as a screen to identify potential synergistic effects of two antibiotics. Two different antibiotic discs were placed about 3 mm apart on the surface of the agar. Plates were incubated overnight in a 37 °C incubator. A larger zone of inhibition between the two discs compared to each drug alone indicated a potential synergistic interaction, which was then further evaluated by the checkerboard assay.

### 2.4. Checkerboard Assays

Checkerboard assays were carried out as described previously [41]. Briefly, two-fold serial dilutions of two different antibiotics were performed in different directions, horizontal and vertical, in a 96-well plate. Diluted cells were added at an OD_600_ of 0.05 and incubated overnight. The OD_600_ was read the following morning. The combinatorial effects were then determined by calculating the fractional inhibitory concentration index (FICI). The FIC (fractional inhibitory concentration) for each drug was determined, which is the concentration of the antibiotic in combination divided by the MIC of the antibiotic alone. The FICI was calculated by the following formula: FICI = (MIC A_A+B_/MIC A) + (MIC B_A+B_/MIC B), where MIC A and MIC B denote the MIC of each antibiotic alone and MIC A_A+B_ and MIC B_A+B_ denote the MICs of the drugs in combination. For a FICI ≤0.5, the combination is synergistic, for a FICI of 0.5–1.0 the combination is additive, and for a FICI >1 and <4, there is no effect. For a FICI ≥4, the interaction is antagonistic. All determinations were made at least two independent times.

### 2.5. Interpretation of Data

If available, the CLSI breakpoint data were used to determine the antimicrobial susceptibility status of the *A. baumannii* strains against most of the standard-of-care antibiotics [18]. The proposed interpretive criteria of *A. baumannii* for rifampin [43] and tigecycline [44] were used for our analysis. Please note that these values do not represent clinical breakpoints as determined by the CLSI. There is no in vitro breakpoint data available for eravacycline, omadacycline, or plazomicin against *A. baumannii* from the CLSI, FDA, or European Committee on Antimicrobial Susceptibility Testing (EUCAST). Therefore, we cannot determine if each strain is susceptible or resistant. However, we used MICs based upon reported standards for tetracyclines and aminoglycosides as a proxy, ≤4.0 μg/mL and ≤8.0 μg/mL as “susceptible”, respectively.

### 2.6. Whole Genome Sequencing

Single colonies were inoculated into 5 mL MHB, and grown overnight at 37 °C, with aeration. DNA was prepared using the DNeasy blood and tissue kit (Qiagen), following manufacturer’s instructions for Gram-negative bacteria. Concentrations of DNA were determined using the Nanodrop 2000 spectrophotometer (ThermoFisher Scientific, Waltham, MA, USA), which ranged from 50–120 ng/μL, with A260–280 ratios between 1.91 and 2.0. Genomc DNA samples were sent to the Rutgers University Genomics Center (Newark, NJ, USA) for whole genome sequencing.

### 2.7. Bioinformatics Analysis

Paired-end sequencing reads (150 bp) were adapter trimmed and quality filtered with the fastp toolkit. Decontamination of human background was performed with Snap-aligner by mapping the reads to a set of human and chimpanzee reference genomes. Microbial reads were assembled with MEGAHIT, and contigs were analyzed with AMRFinderPlus (ver. 3.11) for the presence of antimicrobial resistance genes. An organism-specific single nucleotide polymorphism (SNP) search was also applied by setting the *A. baumannii* flag in AMRFinderPlus.

## 3. Results

### 3.1. Determination of Susceptibilities of Clinical Isolates of A. baumannii to Standard-of-Care Drugs

**Figure 1:**
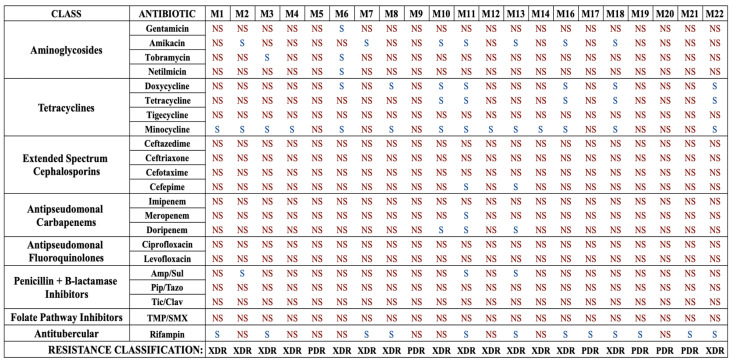
Antibiotic susceptibility profiles of each *A. baumannii* isolate for each standard-of-care antibiotic. Strains are labeled M1–M22. Note that strain M15 was not *A. baumannii* and was excluded from the study. Strains that are susceptible are denoted as S, and those determined to be non-susceptible, as defined as intermediate susceptibility or resistance, are denoted as NS. Each strain is also classified in terms of drug resistance, either XDR or PDR. Amp/Sul: ampicillin–sulbactam; Pip/Tazo: piperacillin–tazobactam, Tic/Clav: ticarcillin–clavulanate, TMP/SMX: trimethoprim–sulfamethoxazole.

To begin, we characterized the susceptibilities of our isolates to a complete panel of 22 antibiotics, including standard-of-care and last-line drugs for the treatment of *A. baumannii* infections, and the antimycobacterial drug rifampin. Standard broth microdilution assays were conducted to determine the MICs and susceptibilities of each strain to each drug (Figure 1 and Table S1). Overall, this collection was highly resistant, with 15 strains (71.4%) being XDR and 6 strains (28.6%) being PDR. Determination of XDR and PDR status was made using current definitions proposed for *Acinetobacter* species [11].

The collection was highly non-susceptible, meaning intermediate or resistant, to the β-lactams (Table 1). High rates of resistance to cephalosporins were also observed, with 95.2% of strains resistant to ceftazidime, 90.5% of strains resistant to cefotaxime and ceftriaxone, and 81.0% resistant to cefepime. In addition, high rates of resistance to β-lactamase inhibitor combination agents were observed, with all strains resistant to piperacillin–tazobactam and ticarcillin–clavulanate, and 85.7% resistant to ampicillin–sulbactam. With regard to the tetracycline class, a majority of strains were resistant to tetracycline (71.4%) and doxycycline (57.2%). Surprisingly, all strains were resistant to tigecycline, the glycylcycline drug typically used as a last-line treatment for MDR *A. baumannii* infections. However, the majority of strains (66.7%) were found to be susceptible to minocycline, a second-generation tetracycline class drug. An overwhelming majority of strains were found to be resistant to carbapenems, with 81.0% resistant to doripenem, 90.5% resistant to meropenem, and all resistant to imipenem. Overall, 85.7% of strains were found to be CRAB, meaning they were resistant to all three carbapenem drugs. Every strain was resistant to the fluoroquinolones ciprofloxacin and levofloxacin and the folate pathway inhibitors trimethoprim/sulfamethoxazole. Finally, this collection also had high resistance rates to the aminoglycosides, with 90.5% resistant to gentamicin, 85.7% resistant to netilmicin, 81.0% resistant to tobramycin, and 61.9% resistant to amikacin. Notably, the antimycobacterial drug rifampin was relatively effective, with 57.1% of strains susceptible. These results suggest that against XDR or PDR strains, rifampin, although not a traditional standard-of-care drug, might be worth adding to the susceptibility testing during clinical laboratory workup.

### 3.2. Determination of Antibiotic Susceptibilities of New Antibiotics

Next, we evaluated the three newer drugs, omadacycline, eravacycline, and plazomicin. Given that all three drugs were recently introduced to the market, there is limited data regarding the potency of each against *A. baumannii* strains, especially against XDR or PDR strains. We determined the MIC values of these drugs against each strain. There are currently no guidelines or standard values regarding clinical breakpoints against *A. baumannii* for any of the three antibiotics, from either the CLSI, FDA, or EUCAST. Therefore, we are unable to determine whether a strain is susceptible or resistant to these drugs. However, we can utilize the MIC values to infer whether any of the antibiotics may be potent, with low MIC values implying susceptibility. We considered an MIC value of ≤4.0 μg/mL to be “susceptible” for omadacycline and eravacycline and ≤8.0 μg/mL to be “susceptible” for plazomicin, based on the CLSI cutoffs for the other tetracyclines or aminoglycosides, respectively. The average MIC values for the three antibiotics against each strain are listed in Table 2. Plazomicin was found to be largely ineffective, exhibiting MIC values >16 against 95.5% of the strains, which likely implies resistance. Plazomicin only exhibited a low MIC value against the strain M3, which was XDR. However, both omadacycline and eravacycline were more effective, with MIC values ≤ 4.0 against 61.9% and 47.6% of strains, respectively. These rates are comparable with those found for minocycline (Table 1). Omadacycline exhibited “susceptible” values against five of six PDR strains, while eravacycline exhibited “susceptible” values against three of six strains, and all MICs were within one dilution except M5, which was within two dilutions. Given the high rates of non-susceptibility against the other standard-of-care tetracycline drugs, these results suggest that minocycline, eravacycline, and omadacycline overcome the tetracycline resistance mechanisms that are present in these strains and could be effective options for treating MDR, XDR, and PDR *A. baumannii* infections.

### 3.3. Determination of Combinatorial Effects of Various Combinations of Antibiotics

As many standard-of-care drugs were largely ineffective against this collection, we next sought to determine if any combinations of drugs would be more effective. We performed disc diffusion assays to screen for potential combinatorial interactions. Two antibiotic discs, each impregnated with antibiotics representing different drug classes, were placed adjacent to each other and visually inspected to determine if a larger zone of inhibition, subjectively estimated as an increase of 2–5 mm, developed between the two discs compared to each drug individually. An observed increased zone of inhibition between the two discs was suggestive of a potential synergistic interaction, which was further evaluated using checkerboard assays. We did not assess all possible antibiotic combinations, instead choosing a representative antibiotic from each drug class for a total of 40 combinations, which are listed Table S2. All combinations with omadacycline and eravacycline were examined, as these drugs were more promising against our collection than plazomicin. For the screening assays, the strain M20 was selected, a PDR isolate that was resistant to every drug tested, although it did exhibit “susceptible” values with omadacycline and eravacycline. Larger zones of inhibition were observed for 13 antibiotic pairings, which are shown in Table 3. No other pairings tested yielded possible combinatorial effects based on visual inspection.

**Figure 2:**
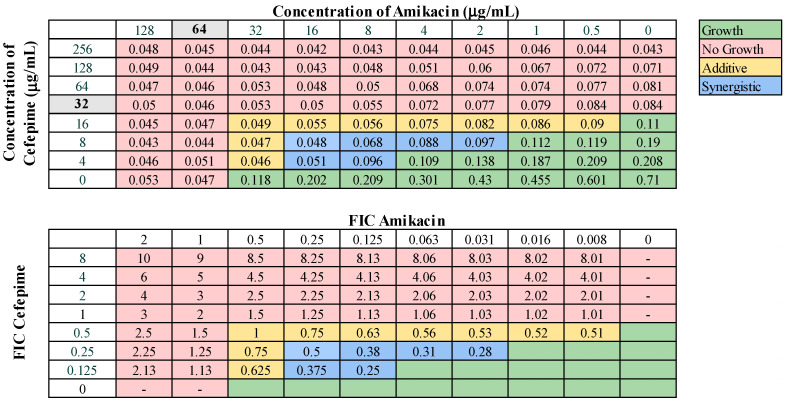
Representative checkerboard assay with amikacin and cefepime against the strain M6. Top: OD_600_ measurements following 16 h of static growth at 37 °C. The MIC values for each drug alone are highlighted in gray. The pink boxes indicate wells in which no bacterial growth occurred (OD_600_ < 0.1), and green boxes indicate wells in which bacterial growth did occur. The box in the bottom right corner contains no drug and serves as a growth control. Bottom: Fractional inhibitory concentrations (FICs) were calculated for each drug (concentration/MIC) and added together for all wells where no growth was observed. The yellow boxes indicate additive interactions (FICI between 0.5–1.0), and blue boxes indicate synergistic interactions (FICI ≤ 0.5).

**Figure 3:**
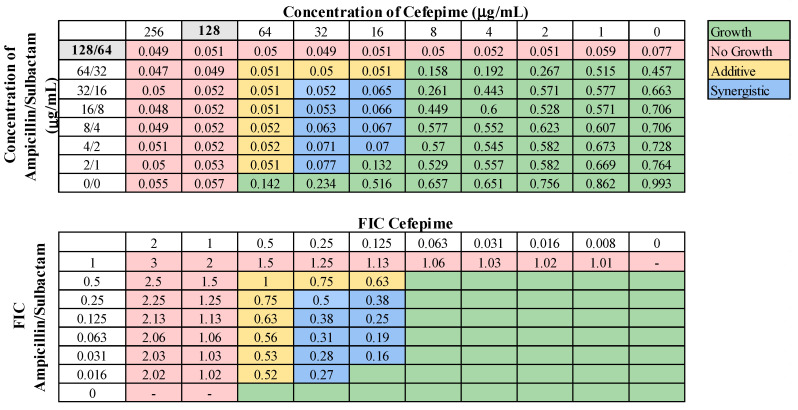
Representative checkerboard assay with cefepime and ampicillin–sulbactam. The PDR strain M20 was grown in the presence of concentration gradients of cefepime and ampicillin–sulbactam, creating various combinations of the two drugs. Top: OD_600_ measurements following 16 h of static growth at 37 °C. The MIC values for each drug alone are highlighted in gray. The pink boxes indicate wells in which no bacterial growth occurred (OD_600_ <0.1), and green boxes indicate wells in which bacterial growth did occur. The box in the bottom right corner contains no drug and serves as a growth control. Bottom: Fractional inhibitory concentrations (FICs) were calculated for each drug (concentration/MIC) and added together for all wells where no growth was observed. The yellow boxes indicate additive interactions (FICI between 0.5–1.0), and blue boxes indicate synergistic interactions (FICI ≤ 0.5).

These 13 combinations were further examined for combinatorial effects against the entire collection using checkerboard assays. The fractional inhibitory concentrations (FIC) for each pair were calculated, as described in Materials and Methods. An FIC index (FICI) value of ≤0.5 for a given antibiotic combination indicated a synergistic interaction, while a FICI of 0.5–1.0 indicated an additive effect. First, we examined combinations of standard-of-care drugs. We found potential interactions among cefepime, amikacin, ampicillin–sulbactam, rifampin, and levofloxacin in various combinations (Table 3). We found that interactions among drugs were not uniform for all strains tested. Of the combinations of standard-of-care antibiotics tested, cefepime combined with rifampin, levofloxacin combined with amikacin, and levofloxacin combined with cefepime did not yield any synergistic interactions. However, levofloxacin in combination with cefepime did result in a high rate (76.2%) of additive effects against these strains. Cefepime in combination with rifampin displayed additive effects against 57.1% of strains. Levofloxacin in combination with amikacin was more ineffective, only exhibiting synergistic effects against 4.8% of strains, additive effects against 23.8% of strains, and no interaction against the rest. We did identify two combinations that might be effective treatment options against highly drug-resistant strains. First, cefepime in combination with amikacin exhibited synergistic effects against 42.9% of strains (Table 3) and additive effects against 38.1%, making this a promising combination for use against highly drug resistant strains. A representative checkerboard assay showing the synergy of this combination against strain M6 is shown in Figure 2. Cefepime paired with ampicillin–sulbactam was the most effective combination, exhibiting synergy in 76.2% and additive effects in 19% of strains. This combination was particularly effective against PDR strains, demonstrating synergistic effects against 83.3% of PDR strains. A representative checkerboard assay showing the synergy between cefepime and ampicillin–sulbactam against strain M20 is shown in Figure 3. Note that none of the combinations tested demonstrated only antagonistic interactions. As we only screened representative drugs from each class, and cefepime combined with amikacin was effective, we analyzed cefepime in combination with another aminoglycoside, tobramycin. We found that 33.3% of strains yielded synergistic effects, with 23.8% resulting in additive effects (Figure S1). This suggests that analyzing additional antibiotics in drug classes that yielded combinatorial effects may reveal additional promising options.

**Figure 4:**
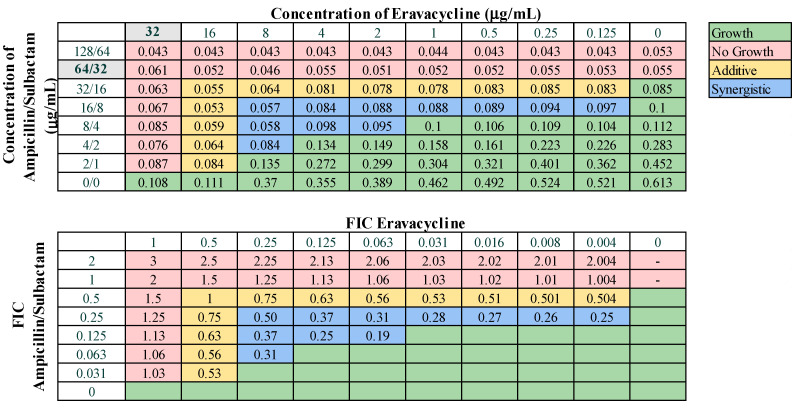
Representative checkerboard assay with eravacycline and ampicillin–sulbactam against strain M8. Top: OD_600_ measurements following 16 h of static growth at 37 °C. The MIC values for each drug alone are highlighted in gray. The pink boxes indicate wells in which no bacterial growth occurred (OD_600_ <0.1), and green boxes indicate wells in which bacterial growth did occur. The box in the bottom right corner contains no drug and serves as a growth control. Bottom: Fractional inhibitory concentrations (FICs) were calculated for each drug (concentration/MIC) and added together for all wells where no growth was observed. The yellow boxes indicate additive interactions (FICI between 0.5–1.0), and blue boxes indicate synergistic interactions (FICI ≤ 0.5).

**Figure 5:**
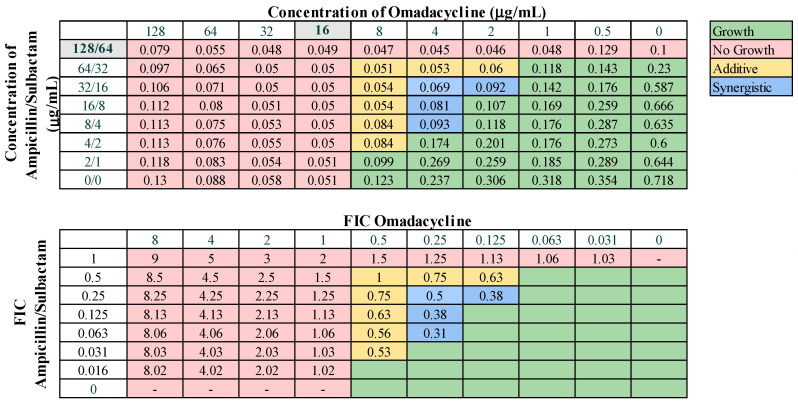
Representative checkerboard assay with omadacycline and ampicillin–sulbactam against strain M21. Top: OD_600_ measurements following 16 h of static growth at 37 °C. The MIC values for each drug alone are highlighted in gray. The pink boxes indicate wells in which no bacterial growth occurred (OD_600_ <0.1), and green boxes indicate wells in which bacterial growth did occur. The box in the bottom right corner contains no drug and serves as a growth control. Bottom: Fractional inhibitory concentrations (FICs) were calculated for each drug (concentration/MIC) and added together for all wells where no growth was observed. The yellow boxes indicate additive interactions (FICI between 0.5–1.0), and blue boxes indicate synergistic interactions (FICI ≤ 0.5).

Next, we assessed combinations of omadacycline or eravacycline with the standard-of-care drugs amikacin, ampicillin–sulbactam, rifampin, and levofloxacin for potential synergistic interactions. Eravacycline was effective in combination with ampicillin–sulbactam, displaying synergy against 38.1% of strains and additive effects against 57.1% of strains (Figure 4). Eravacycline also demonstrated synergy in combination with amikacin against 19.0% of strains and additive effects against 42.9% of strains (Figure S2). Eravacycline in combination with rifampin demonstrated synergy against two strains (9.5%) and additive effects against 85.7% of strains (Figure S3). Eravacycline did not demonstrate any synergistic effects when in combination with levofloxacin. Omadacycline demonstrated synergistic effects against 23.8% of strains when combined with ampicillin–sulbactam (Figure 5) and only 9.5% of strains when combined with levofloxacin (Figure S4). Omadacycline combined with levofloxacin was active against PDR strains, demonstrating synergistic effects against 33.3% and additive effects against the remaining 66.7% strains. Omadacycline in combination with either amikacin or rifampin did not display any synergistic interactions. Omadacycline combined with amikacin demonstrated additive effects against 33.3% of strains, but overall was largely ineffective as a combination. Omadacycline combined with rifampin demonstrated additive effects against 57.1% of strains, and no interaction against 42.8% of strains. However, against the PDR strains, omadacycline combined with rifampin was relatively effective, demonstrating additive effects against 66.7% of strains. Again, none of the combinations involving omadacycline or eravacycline demonstrated antagonistic effects.

### 3.4. Identification of Resistance Genes among A. baumannii Isolates

We next selected eight strains for whole genome sequencing analysis, including three PDR strains (M5, M9, and M20) and five XDR strains with different resistance profiles (M1, M4, M10, M11, and M13). These strains were analyzed for the presence of known antimicrobial resistance genes, as shown in Table 4. The AMEs were widely distributed among the collection, with *aph(6)-Id* and *ant(3*″*)-IIa* present in all strains. All strains possessed at least three different AME genes. In addition, the 16S ribosomal RNA methylase *armA* was present in five strains, which also contributes to aminoglycoside resistance. The presence of *tetB*, which encodes an efflux pump in the major facilitator superfamily (MFS) that extrudes tetracycline drugs, was also found in all eight strains. Numerous genes conferring resistance against β-lactam drugs were identified. Various ADC, Ambler class C β-lactamases that confer resistance against cephalosporins, were identified, including *blaADC-30*, *blaADC-33*, *blaADC-73*, *blaADC-80*, and *blaADC-150*. The cefepime-hydrolyzing enzyme ADC-33 was not widely distributed among our collection, which may explain the synergistic effects found with cefepime and other antibiotics. Five different carbapenem-hydrolyzing, Ambler class D β-lactamase genes were also identified. All strains contained one or more class D enzymes. A mutation in the penicillin-binding protein 3 (PBP3) gene *ftsI* was also present in four strains, which contributes to carbapenem resistance. Strains M10, M11, and M13 are not CRAB strains. M10 possess only OXA-66, which is not enough to confer complete resistance to doripenem or meropenem (Table S1). However, in the presence of an *ftsI* mutation, as in M9, this was enough for high-level resistance to all carbapenems. M11 only contains OXA-94, which confers resistance to imipenem, and M13 contains OXA-66 and OXA-421, which confers resistance to imipenem and meropenem, but not doripenem. All strains contained at least one fluoroquinolone resistance-determining mutation, including an S81L substitution in GyrA and an S84L or E88K substitution in ParC. These mutations are likely present in the entire collection, as 100% of isolates were resistant to both fluoroquinolone drugs (Table 1). Other genes known to confer resistance against macrolides, phenicols, and sulfonamide drugs were also identified. The entire collection was resistant to trimethoprim–sulfamethoxazole, so the *sul1* and *sul2* genes are probably widely distributed. Each of these strains contained more than 10 different resistance genes, which explains why they are so highly drug resistant.

## 4. Discussion

As the spread of antimicrobial resistance among *Acinetobacter* spp. continues to worsen, it has become increasingly imperative to identify which antimicrobial drugs, alone or in combination, might be useful against MDR, XDR, and PDR isolates. We have generated antibiotic susceptibility profiles of 21 clinical isolates of highly drug-resistant *A. baumannii* strains. Commonly used antimicrobials considered standard-of-care in the treatment of *A. baumannii* infections and several newly developed antimicrobials with limited information regarding their effectiveness against *Acinetobacter* species were included in our panel. We then screened and identified synergistic effects between different combinations of these antimicrobials. A striking feature of this collection of strains is the depth of antimicrobial resistance, with all being classified as either XDR or PDR. There were concerning levels of resistance to antimicrobials typically reserved as last-line agents for treating MDR species, such as tigecycline and the carbapenems. These strains are virtually untreatable and infections with such strains are often associated with high mortality rates. These findings further emphasize the urgency in identifying new drugs that are effective against highly drug-resistant *A. baumannii* strains.

Of the standard-of-care antibiotics tested, minocycline was the most effective, with 66.7% of strains found to be susceptible. This finding agrees with our previous findings [41] and others, as minocycline has emerged as a promising alternative for the treatment of CRAB infections [45]. A surveillance study performed in the U.S. demonstrated that overall resistance rates of *Acinetobacter* spp. against minocycline decreased from 56.5% in 2003 to 35.2% in 2012 [15]. In contrast, an inverse pattern was reported from a surveillance study in China, with non-susceptibility rates to minocycline increasing from 13.5% in 2004 to 64.5% in 2014 [46]. This discrepancy is often attributed to which specific efflux pumps were expressed among the *A. baumannii* populations that were evaluated. Widely distributed among our collection was the efflux pump encoded by *tetB*, which is typically associated with resistance against minocycline [47], with a previous study demonstrating that 100% of *A. baumannii* strains lacking *tetB* were susceptible to minocycline [48]. However, we found that all eight of the sequenced strains possess *tetB*, and M1, M4, M10, M11, and M13 were still susceptible to minocycline. The underlying reason for this finding is unknown, but it is possible that some other strain differences that would not be identified in our resistance gene analysis, account for these differences. Minocycline’s high in vitro potency, favorable safety profile, and wide accessibility make it a promising candidate for the treatment of drug-resistant *A. baumannii*. We further emphasize that for *A. baumannii* infections, minocycline should be included in the primary susceptibility screening panel for routine workup in the hospital clinical microbiology lab [41]. In support of this, in their recent 2023 guidelines for treatment of CRAB infections, The Infectious Disease Society of America (IDSA) suggests minocycline as the tetracycline drug of choice in treating CRAB infections [49].

The newest tetracycline drugs available on the market, omadacycline and eravacycline, exhibited low MIC values ≤ 4.0 μg/mL against 61.9% and 47.6% of strains, respectively. Previous research demonstrated that both omadacycline and eravacycline retain activity against bacterial species expressing multiple efflux pumps. Omadacycline was designed with aminomethyl groups at the C7 and C9 positions of the tetracycline D ring, with the C7 modification blocking activity by efflux pumps [50]. Eravacycline contains different modifications, including a single fluorine atom at the C7 position and a pyrrolidineacetamide group at the C9 position [51], which overcomes resistance mechanisms. We found omadacycline to be more potent than eravacycline against our collection, especially against the PDR strains. However, omadacycline is not typically used clinically for the treatment of *A. baumannii* infections. Clinical data regarding the use of omadacycline against *A. baumannii* is very limited, although one recent case study described successful treatment of MDR *A. baumannii* infections in six patients using omadacycline [22]. A prior study comparing the pharmacodynamic responses to omadacycline between *E. coli* and *A. baumannii* found that *A. baumannii* rapidly evolved resistance against omadacycline compared to *E. coli* [52]. Conversely, there is growing data supporting the clinical use of eravacycline, particularly in infections resistant to carbapenems and tigecycline [22]. Nevertheless, omadacycline has an advantage over eravacycline in that it is available in an oral formulation, whereas eravacycline is only available as an intravenous formulation [24]. Further research evaluating omadacycline resistance mechanisms and its activity against highly drug resistant *A. baumannii* is warranted.

We previously examined the potency of eravacycline and omadacycline against an older collection of *A. baumannii* isolates collected from 2004–2012. We found that eravacycline was highly effective against this older collection, which was made up of MDR and XDR *A. baumannii* strains, with low MIC values ≤ 4.0 μg/mL against all isolates. At the time, we found omadacycline to be less effective against the collection, with low MIC values against only 42% of strains [41]. It is worth noting that our current collection, now composed entirely of XDR and PDR strains, displays increased eravacycline MICs but also lower MICs to omadacycline. One in vitro evolution study discovered a unique mechanism of resistance to eravacycline conferred by a deletion mutation in *adeS*, which encodes a sensor kinase that regulates expression of the AdeABC efflux pumps, resulting in overexpression and increased MIC values with eravacycline [53]. It is possible that due to the recent increased usage of eravacycline in clinical settings, *A. baumannii* strains have already begun to evolve new resistance mechanisms against eravacycline.

Another concerning finding was the universal resistance these strains exhibited against tigecycline; a relatively new drug commonly used as a last-resort agent against drug-resistant *A. baumannii*. Increasing resistance of *A. baumannii* to tigecycline has been reported and is most often attributed to various efflux pumps, mainly RND-type pumps such as the multidrug and toxic compound extrusion (MATE) family, the ATP-binding cassette (ABC) transporters, and MFS pumps [54]. Our strains contain TetB, which is an MFS pump, but it is possible that our collection contains additional RND-type pumps, including those not included in our search. The rapid development of widespread resistance to tigecycline is worrisome, and it may foretell the development of similar patterns of resistance against omadacycline or eravacycline in the future.

Our collection exhibited limited susceptibilities to plazomicin. We found that plazomicin is largely ineffective against our collection, with 95.5% of strains exhibiting high MIC values > 8 μg/mL. Our findings are concordant with prior studies that have demonstrated plazomicin’s limited activity against *A. baumannii* [55,56]. In a comparison of in vitro activity, one study found that plazomicin did not exhibit increased activity against *A. baumannii* compared to tobramycin, amikacin, and gentamicin, and also possessed less activity against *A. baumannii* than it did against *Enterobacterales* [26]. In fact, all aminoglycosides tested in this study were largely ineffective as monotherapies, with amikacin exhibiting the lowest resistance rate of 66.7%. The strains demonstrated high resistance rates to gentamicin (95.5%), tobramycin (80.0%), and netilmicin (90.5%). Aminoglycoside resistance is primarily mediated by expression of various AMEs, which catalyze the chemical modification of hydroxyl or amine groups on aminoglycosides to inactivate them. AMEs are classified according to how they chemically modify their substrates and are divided into aminoglycoside acetyltransferases (AACs), aminoglycoside phosphotransferases (APHs), and aminoglycoside nucleotidyltransferases (ANTs) [57]. There were eight different AMEs distributed among our collection, particularly APH(3″)-I, APH(6)-I, and ANT(3″)-II, all of which likely contributed to the high rates of aminoglycoside resistance [58].

Another significant cause of aminoglycoside resistance is the expression of 16S ribosomal RNA methyltransferases (16S RMT). These enzymes methylate 16S rRNA to prevent binding of aminoglycosides to the 30S ribosomal subunit [59]. Many 16S RMT genes have been identified in *A. baumannii,* including *armA,* which was present in five strains (Table 4, [60]). In 2022, in the UK, it was found that 96.5% of clinical isolates of *A. baumannii* harbored at least one 16S RMT gene, most commonly *armA* [61]. Plazomicin, which was primarily designed to overcome resistance due to AMEs, is inherently ineffective against bacteria expressing 16S RMTs and lacks in vitro activity against 16S RMT-expressing *Enterobacterales* [55,62]. The presence of numerous AMEs and *armA* likely conferred the high rates of resistance against aminoglycosides in our collection and suggests that aminoglycoside drugs are ineffective as monotherapies due to widespread distribution of such genes.

Unexpectedly, we found rifampin to be effective against *A. baumannii*, with 57.1% of strains susceptible, in agreement with our previous study with our older collection [41]. Traditionally used as an antimycobacterial agent for tuberculosis infection, rifampin has had minimal clinical use in the treatment of *A. baumannii* infections. Rifampin, like all other rifamycin drugs, selectively inhibits RNA polymerase, thus inhibiting RNA synthesis [63]. As mentioned before, previous in vitro studies have demonstrated synergistic activity when rifampin is combined with other antimicrobials, particularly colistin, but data regarding rifampin as a monotherapy against *A. baumannii* is generally lacking. However, one clinical prospective study found that high dose oral rifampin monotherapy was ineffective in treating ICU patients CRAB infections, as rifampin’s pharmacokinetic properties prevented achievement of adequate therapeutic levels [64]. Pharmacokinetic limitations pose significant obstacles in utilizing rifamycin drugs against *A. baumannii*. An in vitro study also found rifabutin, a rifamycin related to rifampin, to be highly potent against CRAB isolates, primarily at concentrations above 2 mg/L, but these concentrations cannot be feasibly obtained via oral formulations of the drug currently available [65]. Given numerous adverse effects and drug interactions associated with rifamycins, the IDSA currently recommends against the use of rifamycin drugs in treating *A. baumannii* infections [49]. However, if treatment options are severely limited, as with XDR or PDR isolates, perhaps rifamycins could become last-resort drugs. Proper investigations regarding the efficacy of rifamycins against *A. baumannii* are still lacking, and further investigation of rifampin and other rifamycin agents should be performed.

As new drugs are slow to the market, combination therapy may be the most effective option to find new therapeutic strategies quickly. We screened various pairs of drugs to identify those with potential combinatorial effects, and we tested each combination against our entire collection. We found that the combinatorial effects were not universally conserved and displayed strain specific differences. Therefore, physicians must exercise caution when utilizing the literature to find potential combinations to combat highly drug-resistant infections with limited treatment options. This means that there is a chance that a combination will not be effective against the specific patient isolate. It would be prudent for researchers to evaluate multiple strains to determine the potency of the combination to better inform use in clinical settings under emergency situations.

Omadacycline and eravacycline were found to have potent in vitro activity against our collection and were relatively effective when combined with specific antibiotics. Eravacycline was the most effective in combination with ampicillin–sulbactam, exhibiting a relatively high rate (38.1%) of synergistic effects, and demonstrated synergy with amikacin against 19.0% of strains (Figure 4 and Figure S2). Omadacycline was also relatively effective when combined with ampicillin–sulbactam, exhibiting synergy against 23.8% of strains (Figure 5). However, omadacycline was not as effective in combination with other antibiotics. Although prior research is limited, previous studies demonstrated synergistic effects of eravacycline and omadacycline against *A. baumannii*. Eravacycline exhibited synergy in combination with ceftazidime, imipenem, or polymyxin B against CRAB, and we also demonstrated that eravacycline combined with amikacin exhibited synergy against XDR isolates [40,41,66]. Omadacycline has demonstrated synergy in combination with sulbactam, amikacin, or polymyxin B against CRAB isolates [67]. It is possible that the interactions that may exist between these drug pairings are insufficient to completely overcome the extensive resistance mechanisms utilized by our highly resistant strains. Together, these findings suggest that omadacycline and eravacycline could be used in combination with ampicillin–sulbactam against challenging infections.

Our study revealed high rates of synergy exhibited by the combinations of cefepime with ampicillin–sulbactam (76.2%) and cefepime and amikacin (42.9%). None of these three drugs were effective as monotherapies, with high resistance rates among our collection. Clinically, the IDSA already recommends the inclusion of high-dose ampicillin–sulbactam in any combination therapy against CRAB infections [49]. Sulbactam, an irreversible β-lactamase inhibitor traditionally used with ampicillin, binds to β-lactamases to inhibit their activity. However, sulbactam has also been documented in *A. baumannii* to directly bind and saturate penicillin-binding proteins 1 and 3, inhibiting cell wall synthesis [68]. Therefore, the role of sulbactam in treating *A. baumannii* is two-fold, as it complements β-lactam drugs by inhibiting resistance mechanisms and possesses intrinsic antimicrobial activity [68]. In fact, a previous study demonstrated synergy with sulbactam combined with meropenem, tigecycline, colistin, or amikacin against MDR *A. baumannii* [69]. We found a mutation in several strains in PBP3 (*fstI*, Table 4), which is the target of sulbactam [68]. Every strain that contained the *ftsI_A515V* mutation was resistant to ampicillin–sulbactam but displayed synergistic interactions when cefepime was added in combination. Cefepime targets PBP1 and PBP3, and the most common resistance mechanism against it is destruction by β-lactamases [70]. The combination of cefepime and ampicillin–sulbactam includes three separate drugs acting to inhibit cell wall synthesis in *A. baumannii*, possibly overwhelming the different β-lactam resistance mechanisms, allowing for enhanced efficacy. Synergism between cefepime and ampicillin–sulbactam in *A. baumannii* has been reported previously in CRAB isolates [71], and this is the first time synergism between cefepime and ampicillin–sulbactam has been reported for PDR strains. With its high rate of potency, the combination of cefepime and ampicillin–sulbactam should be considered when encountering PDR strains.

We also found high rates of synergy exhibited by cefepime and amikacin combination therapy, with only 19% of strains exhibiting no effects. Synergism has been demonstrated previously in vitro between ampicillin–sulbactam and amikacin in XDR *A. baumannii* strains [38]. Although synergy between β-lactams and aminoglycosides has been reported in vitro, it is important to note that clinical studies have found controversial results. One systematic review of clinical trials including 7863 patients with sepsis found reduced rates of all-cause mortality and clinical failure in patients receiving β-lactam monotherapy compared to those receiving β-lactam–aminoglycoside combination therapy [72]. Furthermore, higher rates of adverse effects, particularly aminoglycoside-associated nephrotoxicity, were seen in combination therapy recipients [72]. These findings highlight potential discrepancies that exist between in vitro and clinical studies, which indicate that physicians would need to weigh the costs to the benefits of using such combinations.

## 5. Conclusions

In this study, we characterized the antibiotic susceptibility profiles of XDR and PDR *A. baumannii* patient isolates. We found that this collection is highly resistant to most or all standard-of-care antibiotics but observed high rates of susceptibility for minocycline and rifampin. We show that the new drugs eravacycline and omadacycline are effective against these strains based on MICs and confirmed that plazomicin is not. We also identified three highly effective antibiotic combinations, cefepime and amikacin, cefepime and ampicillin–sulbactam, and eravacycline and ampicillin–sulbactam. As expected, our strains contained many resistance determinants. Our work suggests that rifampin should be considered as a possible treatment option against highly drug-resistant strains when options are limited. The combinations we identified are promising as possible therapies. However, caution must be utilized, as specific strains may behave differently, and these combinations might not be effective. In a matter of life or death, these options might be worth trying.

## Figures and Tables

**Table 1:** Summary of *A. baumannii* susceptibility to standard-of-care antibiotics.

Antibiotic	Susceptible	Intermediate	Resistant
Gentamicin	4.8%	4.8%	90.5%
Amikacin	33.3%	4.8%	61.9%
Tobramycin	9.5%	9.5%	81.0%
Netilmicin	4.8%	9.5%	85.7%
Doxycycline	33.3%	9.5%	57.1%
Tetracycline	23.8%	4.8%	71.4%
Tigecycline	0.0%	0.0%	100.0%
Minocycline	66.7%	19.0%	14.3%
Ceftazidime	0.0%	4.8%	95.2%
Ceftriaxone	0.0%	9.5%	90.5%
Cefotaxime	0.0%	9.5%	90.5%
Cefepime	9.5%	9.5%	81.0%
Imipenem	0.0%	0.0%	100.0%
Meropenem	4.8%	4.8%	90.5%
Doripenem	14.3%	4.8%	81.0%
Ciprofloxacin	0.0%	0.0%	100.0%
Levofloxacin	0.0%	0.0%	100.0%
Ampicillin–Sulbactam	14.3%	0.0%	85.7%
Piperacillin–Tazobactam	0.0%	4.8%	95.2%
Ticarcillin–Clavulanic Acid	0.0%	0.0%	100.0%
Trimethoprim–Sulfamethoxazole	0.0%	0.0%	100.0%
Rifampin	57.1%	9.5%	33.3%

Interpretive criteria used were determined by the CLSI, when available. The proposed interpretive criteria of *A. baumannii* for rifampin [44] and tigecycline [45] were used for our analysis.

**Table 2:** Average MIC values of eravacycline, omadacycline, and plazomicin (μg/mL).

Strain	Eravacycline	Omadacycline	Plazomicin
M1	8	16	>32
M2	2	12	>32
M3	6	4	6
M4	8	4	>32
M5 *	12	2	>32
M6	16	3	>32
M7	16	8	>32
M8	16	32	>32
M9 *	8	3	>32
M10	3	6	>32
M11	1	0.25	>32
M12	4	1	>32
M13	0.75	0.25	24
M14	4	5	>32
M16	3	2	>32
M17 *	32	16	>32
M18	8	4	32
M19 *	4	2	>32
M20 *	4	2	>32
M21 *	4	2	>32
M22	8	8	>32

* Denotes PDR strains.

**Table 3:** Rates of synergistic, additive, or lack of effects between antibiotic combinations.

Combination	Synergistic	Additive	Indifferent
Cefepime × Ampicillin–Sulbactam	76.2%	19.0%	4.8%
Cefepime × Amikacin	42.9%	38.1%	19.0%
Cefepime × Rifampin	0%	57.1%	42.9%
Levofloxacin × Amikacin	4.8%	23.8%	71.4%
Levofloxacin × Cefepime	9.5%	76.2%	14.3%
Eravacycline × Ampicillin–Sulbactam	38.1%	57.1%	4.8%
Eravacycline × Amikacin	19.0%	42.9%	38.1%
Eravacycline × Rifampin	9.5%	85.7%	4.8%
Eravacycline × Levofloxacin	0%	38.1%	61.9%
Omadacycline × Ampicillin–Sulbactam	23.8%	19.0%	57.1%
Omadacycline × Amikacin	0%	33.3%	66.7%
Omadacycline × Rifampin	0%	57.1%	42.8%
Omadacycline × Levofloxacin	9.5%	47.6%	42.9%

**Table 4:** Identified resistance genes among eight selected *A. baumannii* strains.

Gene Symbol	Class	Description	M1	M4	M5	M9	M10	M11	M13	M20
*aac(3)-Ia*	Aminoglycoside	aminoglycoside N-acetyltransferase AAC(3)-Ia	**X**				**X**		**X**	
*aac(6′)-Ib′*	Aminoglycoside	aminoglycoside N-acetyltransferase AAC(6′)-Ib′					**X**			
*aadA1*	Aminoglycoside	ANT(3*″*)-Ia family aminoglycoside nucleotidyltransferase AadA1	**X**			**X**	**X**		**X**	**X**
*aph(3′)-VIa*	Aminoglycoside	aminoglycoside O-phosphotransferase APH(3′)-VIa	**X**							
*aph(3″)-Ib*	Aminoglycoside	aminoglycoside O-phosphotransferase APH(3*″*)-Ib		**X**	**X**	**X**	**X**	**X**	**X**	**X**
*aph(3′)-Ia*	Aminoglycoside	aminoglycoside O-phosphotransferase APH(3′)-Ia					**X**			
*aph(6)-Id*	Aminoglycoside	aminoglycoside O-phosphotransferase APH(6)-Id	**X**	**X**	**X**	**X**	**X**	**X**	**X**	**X**
*ant(3″)-IIa*	Aminoglycoside	aminoglycoside nucleotidyltransferase ANT(3*″*)-IIa	**X**	**X**	**X**	**X**	**X**	**X**	**X**	**X**
*armA*	Aminoglycoside	16S rRNA (guanine(1405)-N(7))-methyltransferase ArmA	**X**	**X**	**X**	**X**				**X**
*tet(B)*	Tetracycline	tetracycline efflux MFS transporter Tet(B)	**X**	**X**	**X**	**X**	**X**	**X**	**X**	**X**
*blaADC-30*	Beta-lactam	extended-spectrum class C beta-lactamase ADC-30					**X**			
*blaADC-33*	Beta-lactam	cefepime-hydrolyzing class C beta-lactamase ADC-33	**X**							
*blaADC-73*	Beta-lactam	extended-spectrum class C beta-lactamase ADC-73		**X**	**X**	**X**				**X**
*blaADC-80*	Beta-lactam	extended-spectrum class C beta-lactamase ADC-80						**X**		
*blaADC-150*	Beta-lactam	extended-spectrum class C beta-lactamase ADC-150							**X**	
*blaOXA-23*	Beta-lactam	OXA-23 family carbapenem-hydrolyzing class D beta-lactamase OXA-23	**X**	**X**	**X**					**X**
*blaOXA-66*	Beta-lactam	OXA-51 family carbapenem-hydrolyzing class D beta-lactamase OXA-66		**X**	**X**	**X**	**X**		**X**	**X**
*blaOXA-82*	Beta-lactam	OXA-51 family carbapenem-hydrolyzing class D beta-lactamase OXA-82	**X**							
*blaOXA-94*	Beta-lactam	OXA-51 family carbapenem-hydrolyzing class D beta-lactamase OXA-94						**X**		
*blaOXA-421*	Beta-lactam	OXA-213 family carbapenem-hydrolyzing class D beta-lactamase OXA-421							**X**	
*ftsI_A515V*	Beta-lactam	penicillin-binding protein PBP3 FtsI		**X**	**X**	**X**				**X**
*gyrA_S81L*	Flouroquinolone	DNA gyrase subunit A GyrA	**X**	**X**	**X**	**X**	**X**	**X**		**X**
*parC_S84L*	Flouroquinolone	DNA topoisomerase IV subunit A ParC	**X**	**X**	**X**	**X**	**X**		**X**	**X**
*parC_E88K*	Flouroquinolone	DNA topoisomerase IV subunit A ParC						**X**		
*mph(E)*	Macrolide	Mph(E) family macrolide 2′-phosphotransferase	**X**	**X**	**X**	**X**	**X**			**X**
*msr(E)*	Macrolide	ABC-F type ribosomal protection protein Msr(E)	**X**	**X**	**X**	**X**				**X**
*catB8*	Phenicol	type B-3 chloramphenicol O-acetyltransferase CatB8					**X**			
*floR*	Phenicol	chloramphenicol/florfenicol efflux MFS transporter FloR						**X**		
*sul1*	Sulfonamide	sulfonamide-resistant dihydropteroate synthase Sul1	**X**			**X**	**X**		**X**	**X**
*sul2*	Sulfonamide	sulfonamide-resistant dihydropteroate synthase Sul2	**X**	**X**	**X**	**X**	**X**	**X**	**X**	**X**

## Data Availability

All sequencing data is available on the GeoSeeq platform, a publicly accessible database connecting researchers with tools for public health surveillance (https://portal.geoseeq.com/sample-groups/63d25b1e-8039-4b1d-bbb8-ab461d436055).

## Supplementary Materials

The following supporting information can be downloaded at: https://www.mdpi.com/article/10.3390/microorganisms12071353/s1, Figure S1: Representative checkerboard assay with the combination of cefepime and tobramycin; Figure S2: Representative checkerboard assay with the combination of eravacycline and amikacin; Figure S3: Representative checkerboard assay with eravacycline and rifampin; Figure S4: Representative checkerboard assay with omadacycline and levofloxacin; Table S1: MIC values for all drugs against A. baumannii patient isolates; Table S2: List of combinations screened by disc diffusion assay.
