# Supplementary material for: Combinations of Antibiotics Effective against Extensively- and Pandrug-Resistant Acinetobacter baumannii Patient Isolates
Source: Microorganisms. 2024 Jul 2;12(7):1353. doi: 10.3390/microorganisms12071353 (PMC11279082; doi:10.3390/microorganisms12071353)
Supplement: Supplementary file 1 [file microorganisms-12-01353-s001.zip › Halim et al. supplemental figures_revised.pdf]

Combinations of antibiotics effective against Extensively- and Pandrug Resistant *Acinetobacter baumannii* patient isolates

Justin Halim, Rachel A. Carr, Rebecca Fliorent, Keertana Jonnalagadda, Maftuna Kurbonnazarova, Muskanjot Kaur, Ian Millstein, and Valerie J. Carabetta[1]

Contents

Table S2: List of combinations screened by disc diffusion assay. 2

Figure S1: Representative checkerboard assay with cefepime and tobramycin......................... 3

Figure S2: Representative checkerboard assay with amikacin and eravacycline....................... 4

Figure S3: Representative checkerboard assay with eravacycline and rifampin. 5

Figure S4: Representative checkerboard assay with omadacycline and levofloxacin. 6

Table S2: List of combinations screened by disc diffusion assay.

Antibiotic Combination	Synergy Observed
Amikacin x Eravacycline	Yes
Amikacin x Cefepime	Yes
Amikacin x Meropenem	No
Amikacin x Levofloxacin	Yes
Amikacin x Amp/Sul	No
Amikacin x TMP/SMX	No
Amikacin x Rifampin	No
Amikacin x Doxycycline	No
Eravacycline x Cefepime	No
Eravacycline x Meropenem	No
Eravacycline x Levofloxacin	Yes
Eravacycline x Amp/Sul	Yes
Eravacycline x TMP/SMX	No
Eravacycline x Rifampin	Yes
Omadacycline x Cefepime	No
Omadacycline x Meropenem	No
Omadacycline x Levofloxacin	Yes
Omadacycline x Amp/Sul	Yes
Omadacycline x TMP/SMX	No
Omadacycline x Rifampin	Yes
Cefepime x Meropenem	No
Cefepime x Levofloxacin	Yes
Cefepime x Amp/Sul	Yes
Cefepime x TMP/SMX	No
Cefepime x Rifampin	Yes
Cefepime x Doxycycline	No
Meropenem x Levofloxacin	No
Meropenem x Amp/Sul	No
Meropenem x TMP/SMX	No
Meropenem x Rifampin	No
Meropenem x Doxycycline	No
Levofloxacin x Amp/Sul	Yes
Levofloxacin x TMP/SMX	No
Levofloxacin x Rifampin	No
Levofloxacin x Doxycycline	No
Amp/Sul x TMP/SMX	No
Amp/Sul x Rifampin	No
Amp/Sul x Doxycycline	No
TMP/SMX x Rifampin	No
TMP/SMX x Doxycycline	No

Concentration of Cefepime (µg/mL)

Concentration of Tobramycin (µg/mL)	256	128	64	**32**	16	8	4	2	1	0
128	0.099	0.099	0.091	0.098	0.131	0.102	0.097	0.09	0.076	0.096
64	0.084	0.089	0.091	0.085	0.107	0.087	0.082	0.078	0.081	0.083
32	0.086	0.084	0.092	0.088	0.086	0.087	0.09	0.081	0.083	0.084
16	0.077	0.062	0.062	0.062	0.063	0.07	0.064	0.061	0.06	0.075
**8**	0.067	0.064	0.068	0.068	0.067	0.067	0.069	0.072	0.068	0.079
4	0.069	0.071	0.072	0.07	0.078	0.069	0.072	0.084	0.086	0.104
2	0.075	0.07	0.078	0.075	0.082	0.081	0.091	0.09	0.253	0.596
0	0.068	0.079	0.079	0.086	0.101	0.106	0.107	0.188	0.271	0.854

Legend
Growth
No Growth
Additive
Synergistic

FIC Cefepime

FIC Tobramycin	8	4	2	1	0.5	0.25	0.125	0.063	0.031	0
16	24	20	18	17	16.5	16.25	16.13	16.06	16.03	-
8	16	12	10	9	8.5	8.25	8.13	8.06	8.03	-
4	12	8	6	5	4.5	4.25	4.13	4.06	4.03	-
2	10	6	4	3	2.5	2.25	2.13	2.06	2.03	-
1	9	5	3	2	1.5	1.25	1.13	1.06	1.03	-
0.5	8.5	4.5	2.5	1.5	1	0.75	0.63	0.56	0.53	
0.25	8.25	4.25	2.25	1.25	0.75	0.5	0.37	0.31		
0	-	-	-	-						

Figure S1: Representative checkerboard assay with cefepime and tobramycin against strain M13. Top: OD_{600} measurements following 16 hours of static growth at 37°C. The MIC values for each drug alone are highlighted in gray. The pink boxes indicate wells in which no bacterial growth occurred ($OD_{600} < 0.1$), and green boxes indicate wells in which bacterial growth did occur. The box in the bottom right corner contains no drug and serves as a growth control. Bottom: Fractional inhibitory concentrations (FICs) were calculated for each drug (concentration/MIC) and added together for all wells where no growth was observed. The yellow boxes indicate additive interactions (FICI between 0.5-1.0), and blue boxes indicate synergistic interactions (FICI ≤ 0.5).

Concentration of Amikacin (µg/mL)

Concentration of Eravacycline (µg/mL)	128	64	32	16	8	**4**	2	1	0.5	0
32	0.044	0.042	0.042	0.042	0.045	0.044	0.049	0.05	0.047	0.053
16	0.044	0.042	0.045	0.043	0.045	0.047	0.047	0.049	0.049	0.049
**8**	0.048	0.042	0.045	0.046	0.047	0.047	0.045	0.05	0.052	0.073
4	0.051	0.045	0.043	0.046	0.046	0.048	0.048	0.057	0.078	0.129
2	0.048	0.045	0.044	0.046	0.047	0.047	0.058	0.088	0.16	0.217
1	0.045	0.043	0.045	0.044	0.046	0.046	0.055	0.198	0.201	0.283
0.5	0.046	0.045	0.044	0.044	0.045	0.045	0.046	0.19	0.219	0.353
0	0.045	0.045	0.046	0.047	0.05	0.051	0.168	0.312	0.557	0.757

Legend
Growth
No Growth
Additive
Synergistic

FIC Amikacin

FIC Eravacycline	32	16	8	4	2	1	0.5	0.25	0.125	0
4	36	20	12	8	6	5	4.5	4.25	4.13	-
2	34	18	10	6	4	3	2.5	2.25	2.13	-
1	33	17	9	5	3	2	1.5	1.25	1.13	-
0.5	32.5	16.5	8.5	4.5	2.5	1.5	1	0.75	0.63	
0.25	32.25	16.25	8.25	4.25	2.25	1.25	0.75	0.5		
0.125	32.13	16.13	8.13	4.13	2.13	1.13	0.51			
0.063	32.06	16.06	8.06	4.06	2.06	1.06	0.56			
0	-	-	-	-	-	-				

Figure S2: Representative checkerboard assay with amikacin and eravacycline against strain M2. Top: OD_{600} measurements following 16 hours of static growth at 37°C. The MIC values for each drug alone are highlighted in gray. The pink boxes indicate wells in which no bacterial growth occurred ($OD_{600} < 0.1$), and green boxes indicate wells in which bacterial growth did occur. The box in the bottom right corner contains no drug and serves as a growth control. Bottom: Fractional inhibitory concentrations (FICs) were calculated for each drug (concentration/MIC) and added together for all wells where no growth was observed. The yellow boxes indicate additive interactions (FICI between 0.5-1.0).

Concentration of Eravacycline (μg/mL)

Concentration of Rifampin (μg/mL)	**4**	2	1	0.5	0.25	0.125	0.0625	0.03125	0.01563	0
32	0.044	0.045	0.045	0.044	0.044	0.044	0.047	0.045	0.044	0.06
**16**	0.047	0.047	0.051	0.054	0.055	0.06	0.058	0.064	0.063	0.061
8	0.051	0.052	0.056	0.083	0.143	0.117	0.116	0.135	0.125	0.129
4	0.046	0.053	0.096	0.158	0.149	0.192	0.155	0.185	0.165	0.211
2	0.055	0.063	0.321	0.394	0.405	0.423	0.429	0.454	0.377	0.424
1	0.047	0.25	0.363	0.451	0.389	0.321	0.394	0.509	0.387	0.458
0.5	0.048	0.258	0.452	0.524	0.395	0.504	0.532	0.586	0.547	0.59
0	0.068	0.66	0.702	0.696	0.665	0.726	0.795	0.907	0.805	0.948

Growth
No Growth
Additive
Synergistic

FIC Eravacycline

FIC Rifampin	**1**	0.5	0.25	0.125	0.063	0.031	0.016	0.008	0.004	0
2	3	2.5	2.25	2.13	2.06	2.03	2.02	2.01	2.004	-
**1**	2	1.5	1.25	1.13	1.06	1.03	1.02	1.01	1.004	-
0.5	1.5	1	0.75	0.63						
0.25	1.25	0.75	0.5							
0.125	1.13	0.63								
0.063	1.06									
0.031	1.03									
0	-									

Figure S3: Representative checkerboard assay with eravacycline and rifampin against strain M22. Top: OD_{600} measurements following 16 hours of static growth at 37°C. The MIC values for each drug alone are highlighted in gray. The pink boxes indicate wells in which no bacterial growth occurred (OD_{600} < 0.1), and green boxes indicate wells in which bacterial growth did occur. The box in the bottom right corner contains no drug and serves as a growth control. Bottom: Fractional inhibitory concentrations (FICs) were calculated for each drug (concentration/MIC) and added together for all wells where no growth was observed. The yellow boxes indicate additive interactions (FICI between 0.5-1.0), and blue boxes indicate synergistic interactions (FICI ≤ 0.5).

Concentration of Levofloxacin (µg/mL)

Concentration of Omadacycline (µg/mL)	64	32	**16**	8	4	2	1	0.5	0.25	0
128	0.064	0.066	0.06	0.058	0.084	0.094	0.094	0.062	0.062	0.064
64	0.065	0.056	0.059	0.058	0.061	0.059	0.061	0.062	0.062	0.064
32	0.058	0.051	0.055	0.057	0.062	0.058	0.056	0.055	0.053	0.053
**16**	0.051	0.049	0.054	0.055	0.119	0.084	0.089	0.078	0.074	0.068
8	0.048	0.049	0.056	0.057	0.132	0.138	0.13	0.122	0.119	0.127
4	0.05	0.049	0.056	0.065	0.07	0.242	0.267	0.253	0.233	0.219
2	0.051	0.048	0.057	0.187	0.086	0.327	0.257	0.24	0.272	0.279
0	0.054	0.048	0.062	0.319	0.487	0.349	0.695	0.674	0.697	0.706

Growth
No Growth
Additive
Synergistic

FIC Levofloxacin

FIC Omadacycline	4	2	1	0.5	0.25	0.125	0.063	0.031	0.016	0
8	12	10	9	8.5	8.25	8.13	8.06	8.03	8.02	-
4	8	6	5	4.5	4.25	4.13	4.06	4.03	4.02	-
2	6	4	3	2.5	2.25	2.13	2.06	2.03	2.02	-
2	5	3	2	1.5	1.25	1.13	1.06	1.03	1.02	-
0.5	4.5	2.5	1.5	1						
0.25	4.25	2.25	1.25	0.75	0.5					
0.125	4.13	2.13	1.13		0.38					
0	-	-	-							

Figure S4: Representative checkerboard assay with omadacycline and levofloxacin against strain M17. Top: OD_{600} measurements following 16 hours of static growth at 37°C. The MIC values for each drug alone are highlighted in gray. The pink boxes indicate wells in which no bacterial growth occurred (OD_{600} < 0.1), and green boxes indicate wells in which bacterial growth did occur. The box in the bottom right corner contains no drug and serves as a growth control. Bottom: Fractional inhibitory concentrations (FICs) were calculated for each drug (concentration/MIC) and added together for all wells where no growth was observed. The yellow boxes indicate additive interactions (FICI between 0.5-1.0), and blue boxes indicate synergistic interactions (FICI ≤ 0.5).
